# Left hippocampus sparing model for glioblastoma radiotherapy by utilizing knowledge‐based planning and multi‐criteria optimization

**DOI:** 10.1002/acm2.70014

**Published:** 2025-02-15

**Authors:** Shima Y. Tari, Amr Heikal, Connie Le, Fan Yang, Deepak Dinakaran, John Amanie, Albert Murtha, Lindsay S. Rowe, Wilson H. Roa, Samir Patel

**Affiliations:** ^1^ Department of Medical Physics Cross Cancer Institute Edmonton Alberta Canada; ^2^ Department of Oncology Division of Medical Physics University of Alberta Edmonton Alberta Canada; ^3^ Division of Radiation Oncology Department of Oncology University of Alberta Edmonton Alberta Canada; ^4^ Present address: Mayo Clinic Department of Radiation Oncology Phoenix AZ USA; ^5^ Present address: Sunnybrook Health Science Center Department of Radiation Oncology Toronto Ontario Canada

**Keywords:** deep learning, glioblastoma, knowledge‐based planning, multi‐criteria optimization, radiation therapy planning

## Abstract

**Purpose:**

Results of a prospective, randomized controlled trial at our institute demonstrate an association between the dose to the left hippocampus and neurocognitive decline post‐radiotherapy for patients with glioblastoma. To minimize the dose to the left hippocampus, a left hippocampus sparing model was created using RapidPlan (RP) and multi‐criteria optimization (MCO).

**Materials and methods:**

For 147 patients with newly diagnosed glioblastoma treated with volumetric modulated arc therapy (VMAT), the left and right hippocampus were delineated. Ninety‐seven of 147 VMAT plans were used to configure a RP model named HCS1. The remaining 50 VMAT plans were used for the model validation. All 97 plans were replanned with the HCS1 and further optimized using MCO (HCS1+MCO). MCO was used to explore the trade‐off between reducing the left hippocampus mean dose and planning objectives for the targets and other organs‐at‐risk (OAR) for HCS1 plans. These plans were used to create a new model called HCS2. MCO and RP model configuration were done within the Eclipse treatment planning system.

**Results:**

The final HCS2 model decreased the mean dose to the left hippocampus by 26% compared to clinically treated plans without reducing target coverage for 50 validation data. The mean dose to the left hippocampus decreased from 32.65 Gy in clinically treated plans, 30.45 Gy in HCS1‐generated plans, and 24.04 Gy in HCS2‐generated plans. The mean volume receiving 95% of the prescription dose (V95%) of the planning target volume was 99.08% ± 1.39% in clinically treated plans, 99.03% ± 1.37% in HCS1‐generated plans, and 98.80% ± 1.48% in HCS2‐generated plans. Mean dose to 0.1 cc of the brainstem improved from 45.91 Gy in clinically treated plans to 39.29 Gy in HCS2‐generated plans.

**Conclusions:**

The RP model and MCO helps to decrease left hippocampus mean dose while maintaining the target volume coverage and OAR sparing comparable to clinically treated plans for glioblastoma patients.

## 1 INTRODUCTION

Glioblastoma is the most common primary malignant brain tumor in adults.^1^ Standard management consists of maximal safe resection followed by postoperative radiotherapy (RT) of 60 Gy in 30 fractions with concurrent and maintenance temozolomide chemotherapy.^2^ Prognosis following standard therapy remains dismal, with median survival of less than 2 years. However, about 20% of patients survive beyond 5 years, making radiation‐induced impact on neurocognitive function and quality of life important considerations of modern clinical practice. Irradiation of the hippocampus, which has important roles in memory and learning, is associated with neurocognitive decline after RT and hippocampal avoidance can help preserve memory and quality of life.^3^ A mean left hippocampal dose greater than 30 Gy is associated with neurocognitive decline, including visual and verbal memory deficits, in young patients with brain tumors treated with RT.^4^, ^5^

In a randomized controlled trial for adults with newly diagnosed glioblastoma at our institution, we reported an association of hippocampal dose with neurocognitive decline after RT.^6^ One hundred thirty‐three patients aged 18 to 70 years old underwent maximal safe resection and were randomized to either 60 Gy in 30 fractions over 6 weeks (conventional RT) or 60 Gy in 20 fractions over 4 weeks (short‐course RT), with temozolomide chemotherapy in both arms. The mean left hippocampal dose was associated with worsened cognitive impairment after RT (*p* = 0.005).

RapidPlan (Varian Medical Systems, Inc., Palo Alto, CA; RP) is a commercially available knowledge‐based planning tool that uses data from previously treated patients to estimate the dose volume histogram (DVH) for a new patient. The algorithm correlates the geometric location of the targets with respect to that of the OARs to the associated dosimetry.^7^, ^8^ It uses the structure sets, dose prescriptions, and field geometries of existing patient plans to predict a range of achievable OAR DVHs for a new patient, and these DVH estimates can be used to generate optimization objectives. RP has been used increasingly in recent years for several sites,^9^ which helps to standardize patient treatments by reducing inconsistency and variability in treatment planning.^10^, ^11^, ^12^

Another optimization tool within the Varian's Eclipse treatment planning system (TPS) is multi‐criteria optimization (MCO). This tool explores a Pareto surface for the selection of optimal plans^13^, ^14^, ^15^ and generates a set of the plans to explore the trade‐off between different objectives to choose the best plan that meets the clinical goals. The combination of RP and MCO has been shown to improve the treatment plan quality.^16^, ^17^

RP has been used to generate a treatment planning model for patients with glioblastoma that has been shown to increase planning efficiency compared to manual treatment planning.^18^ In this study, we used RP in combination with MCO to create a model that generates treatment plans for patients with glioblastoma to reduce the dose received by the left hippocampus. Our aims were to use MCO to explore the limit to which the dose to the left hippocampus could be reduced while maintaining the plan quality at a clinically acceptable level compared to clinically treated plans, and compare dose parameters for differences in the PTV, left hippocampus, and brainstem of the final RP in combination with MCO plans and clinically treated plans.

## 2 METHODS

### 2.1 Patient data

One hundred and forty‐seven patients were included in this planning study. The patients were treated with conventionally fractionated (60 Gy in 30 fractions) as per standard of care or on a randomized controlled trial^6^ or hypofractionated, short‐course (60 Gy in 20 fractions) radiotherapy on the same randomized controlled trial during the trial. Target volumes and OARs were contoured using a computed tomography (CT) simulation scan co‐registered to a postoperative, diagnostic magnetic resonance imaging (MRI) scan with gadolinium contrast enhancement as previously described.^6^ The planning target volume (PTV) was defined by adding a 5 mm three‐dimensional margin to the clinical target volume. The hippocampus was delineated using established guidelines established by Gondi et al.^19^ for a planned exploratory analysis of periventricular structure dosimetry.^6^ All patients were planned using the Varian volumetric modulated arc therapy (VMAT) solution RapidArc with 6 MV photons. Three noncoplanar arcs are used for most of the plans, as well as one full arc and two half‐arcs. For the current study, the 18 patients who had their left hippocampus partially resected at the time of surgery and eight patients with outlier data from the randomized controlled trial were excluded from this planning study.

For the PTV, the protocol required that 95% of the PTV was to be covered by 60 Gy. The allowable dose within the PTV was between 57 Gy (95% of 60 Gy) and 66 Gy (110% of 60 Gy). A maximum dose allowed to > 1% of the PTV was 66 Gy. The protocol dose constraints for the OARs are shown in Table 1. A dose constraint for the hippocampus was not specified in the protocol for enrolled patients and subsequently optimization objectives were not used for the left hippocampus in the preparation of clinically treated plans. Based on published literature,^6^ a mean dose of less than 30 Gy was used as the dose constraint for the left hippocampus in the current study. The PTV coverage was allowed to be compromised to meet the maximum dose limits of the OARs, where the maximum dose refers to the dose to 1% of each volume.

**TABLE 1:** Dose constraints for critical structures.

Critical structures	Maximum dose	
	Conventional	Hypofractionated
Brainstem	60 Gy	53 Gy
Optic chiasm	56 Gy	50 Gy
Optic nerves	55 Gy	49 Gy
Eyes	50 Gy	45 Gy
Lenses	7 Gy	7 Gy
Spinal cord	54 Gy	46 Gy

### 2.2 Planning—RP and MCO

We prioritized the use of the trial data towards training the glioblastoma RP model; our model was trained with a pragmatic approach using a broad range of brain sites, PTV volumes, and both standard and short‐course dose prescriptions so that the final model could provide sufficient plan quality for clinical use in a wide range of clinical scenarios. Therefore, RP was used to create a model using 97 of the 107 plans in our trial dataset. The model was configured using 39 conventional plans and 58 hypofractionated plans. Fifty remaining plans (10 within trial and 40 outside trial) in the dataset were used to evaluate the model including 43 conventional plans and seven hypofractionated plans. The RP model was used to generate DVH estimates for the brainstem, cerebrum, chiasm, cochleae, cord, eyes, the left and right hippocampus, lenses, and optic nerves. A structure called PTV_opt was used for optimization and evaluation when there was an overlap between the PTV and the OARs with the PTV_opt being cropped from those critical structures. The same PTV_opt structure was used for our model training and the optimization objective for the target.

The RP model was generated in two steps: data extraction and model training, where the model was refined by an iterative process to remove the outliers.^20^ The plan quality of the data used for creating an RP model is important in generating a good quality plan for a new patient. In this work, another approach was taken to refine our model by using the report generated through the Model Analytics tool from Varian. This report provides suggestions to improve the model either by excluding data or adding data where there is a gap in data. Using the Model Analytics report, the recommended plans with outlier structures were evaluated and removed from the data as deemed appropriate. The refined data was trained for a model named HCS1. This model was then used in combination with MCO to further improve the quality of the plans, which were then used to create a second version of the model.

**FIGURE 1:**
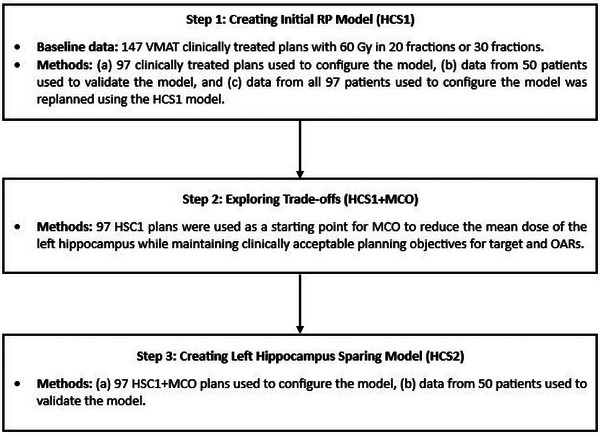
Summary of the process to create a left hippocampus sparing model.

The 97 plans were replanned using the HCS1 model‐generated optimization objectives and were further optimized using MCO (HCS1+MCO). MCO was used to explore the trade‐off between reducing the left hippocampus mean dose and the planning objectives for targets. The upper and lower doses, and the homogeneity of the PTV were among the optimization objectives selected for trade‐off exploration as homogeneity of the PTV helps reduce the hotspots and increase minimum dose coverage.^20^ The brainstem added to the trade‐off exploration because of its proximity to the hippocampus. After calculating a set of plans based on the chosen objectives, a slider bar is displayed for each objective in the MCO workspace. Each slider bar can be moved to investigate trade‐offs where the manipulation of one slider affects the slider bars of the other objectives. There is also an option to restrict a slider bar from further exploration if needed. For our case, the left hippocampus mean dose slider was moved as low as possible without compromising target objectives. As soon as one of the target objectives started to fail, it was restricted, and the left hippocampus slider bar was pushed more if possible. This iterative process was done until all target objectives were restricted. Since the clinical plans did not have any optimization objectives to minimize the mean dose to the left hippocampus, using RP and MCO enabled us to improve the quality of plans to spare left hippocampus while maintaining original planning objectives. The HCS1+MCO plans were used to create a new left hippocampus sparing model called HCS2. The same data and method as HCS1 were used for training and validation. The summary of the approach is shown in Figure 1.

Through an iterative process, the finalized optimization objectives were automatically generated by a RP model. The DVH estimate predicted by the RP model was used as the line objective for OARs. An upper dose constraint was added to the OARs optimization objectives following our protocol (Table 1). The priority and percentage for PTV optimization objectives were finalized after exploring different combinations of optimization objectives to plan our validation data. Table 2 shows the final optimization objectives that were used for the HCS2 model. Table 3 shows the normal tissue objectives (NTOs) used in the optimization.

**TABLE 2:** Final optimization objectives and priorities used for the HCS2 model.

Structure	Type	Volume (%)	Dose	Priority
PTV	Upper	0	103.3%	160
	Lower	100	102.5%	160
Brainstem	Upper	0	53 Gy	Generated
	Line	Generated	Generated	Generated
Cerebrum	Line	Generated	Generated	Generated
Chiasm	Upper	0	50 Gy	Generated
	Line	Generated	Generated	Generated
Cochlea (Left and right)	Line	Generated	Generated	Generated
Cord	Upper	0	46 Gy	Generated
	Line	Generated	Generated	Generated
Eye (Left and right)	Upper	0	45 Gy	Generated
	Line	Generated	Generated	Generated
Hippocampus (Left and right)	Line	Generated	Generated	Generated
Lens (Left and right)	Upper	0	7 Gy	Generated
	Line	Generated	Generated	Generated
Optic nerve (Left and right)	Upper	0	49 Gy	Generated
	Line	Generated	Generated	Generated

Abbreviation: PTV, planning target volume.

**TABLE 3:** Normal tissue objectives used for the HCS2 model.

NTO parameters	Value
Priority	130
Distance from target border	0.3 cm
Start dose (%)	97
End dose (%)	50
Fall‐off	0.2

Abbreviation: NTOs, normal tissue objectives.

MCO and RP model configuration were done within the Eclipse treatment planning system (version 15.6).

The hippocampus is not contoured routinely at all clinics for treatment planning of patients with glioblastoma, so no optimization objective is set for the hippocampus in such cases. We investigated if the final HCS2 model would lower the dose to the hippocampus when applied to a clinical case, even without use of a hippocampus optimization objective to improve generalizability of our results. For this purpose, the HCS2 RP model was used to optimize the dose distribution for 50 validation data.

### 2.3 Statistical analysis

The final plans (HCS2 plans) for validation data were assessed by a radiation oncologist to assess them for clinical acceptability. For each patient, dose parameters for the PTV, left hippocampus, and brainstem were evaluated in the set of the clinically treated, the HCS1 and the HCS2 plans. The volume receiving 95% of the prescription dose (V95%), dose to 0.1 cc of the volume, and maximum dose were assessed for the PTV. The mean dose was assessed for the left hippocampus and dose to 0.1 cc was assessed for the brainstem. The number of monitor units (MUs) were compared. Differences between the set of three plans for each patient were evaluated using the Wilcoxon matched‐pairs signed‐rank test.

For all statistical comparisons, a significance level of 0.05 was used. Statistical analysis was done using Microsoft Excel (Microsoft Corp., Redmond, WA), version 2309.

## 3 RESULTS

**FIGURE 2:**
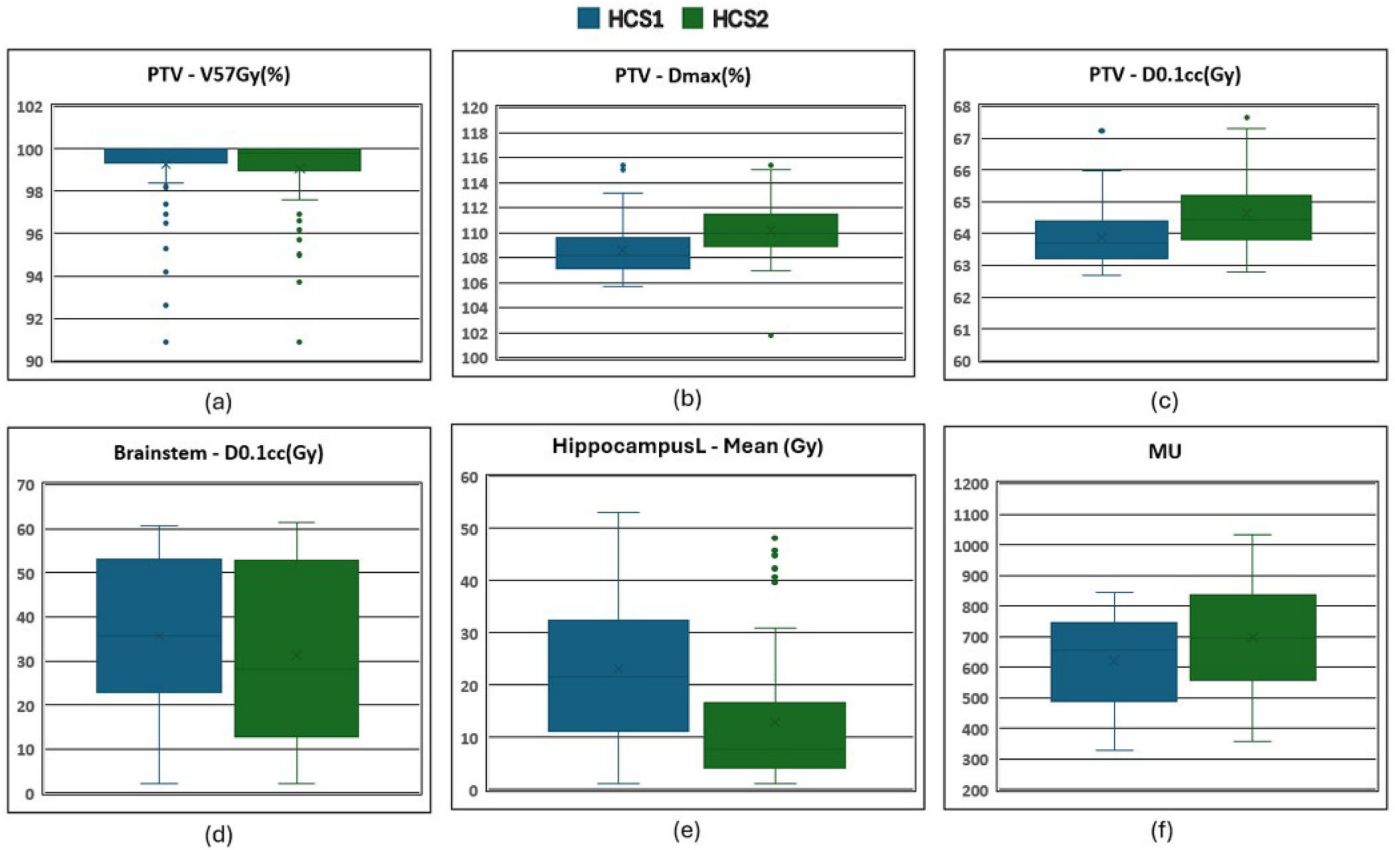
Box and Whisker plots for 97 plans used to configure the RP models (a) V_57Gy_ (in %) for PTV, (b) maximum dose of PTV, (c) D_0.1cc_ (in Gy) for PTV, (d) D_0.1cc_ (in Gy) for brainstem, (e) mean dose for left hippocampus, and (f) Total MU delivered for HCS1 (blue), and HCS2 (green) models. MU, monitor unit; PTV, planning target volume; RP, RapidPlan.

The final left hippocampus sparing HCS2 model was successfully developed using 97 HCS1+MCO plans and validated using data from 50 patients. Figure 2 shows different metrics for data used to create the HCS1 and HCS2 models. The mean dose to the left hippocampus has dropped by 44% for data used to configure the HCS2 model compared to the HCS2 model.

**FIGURE 3:**
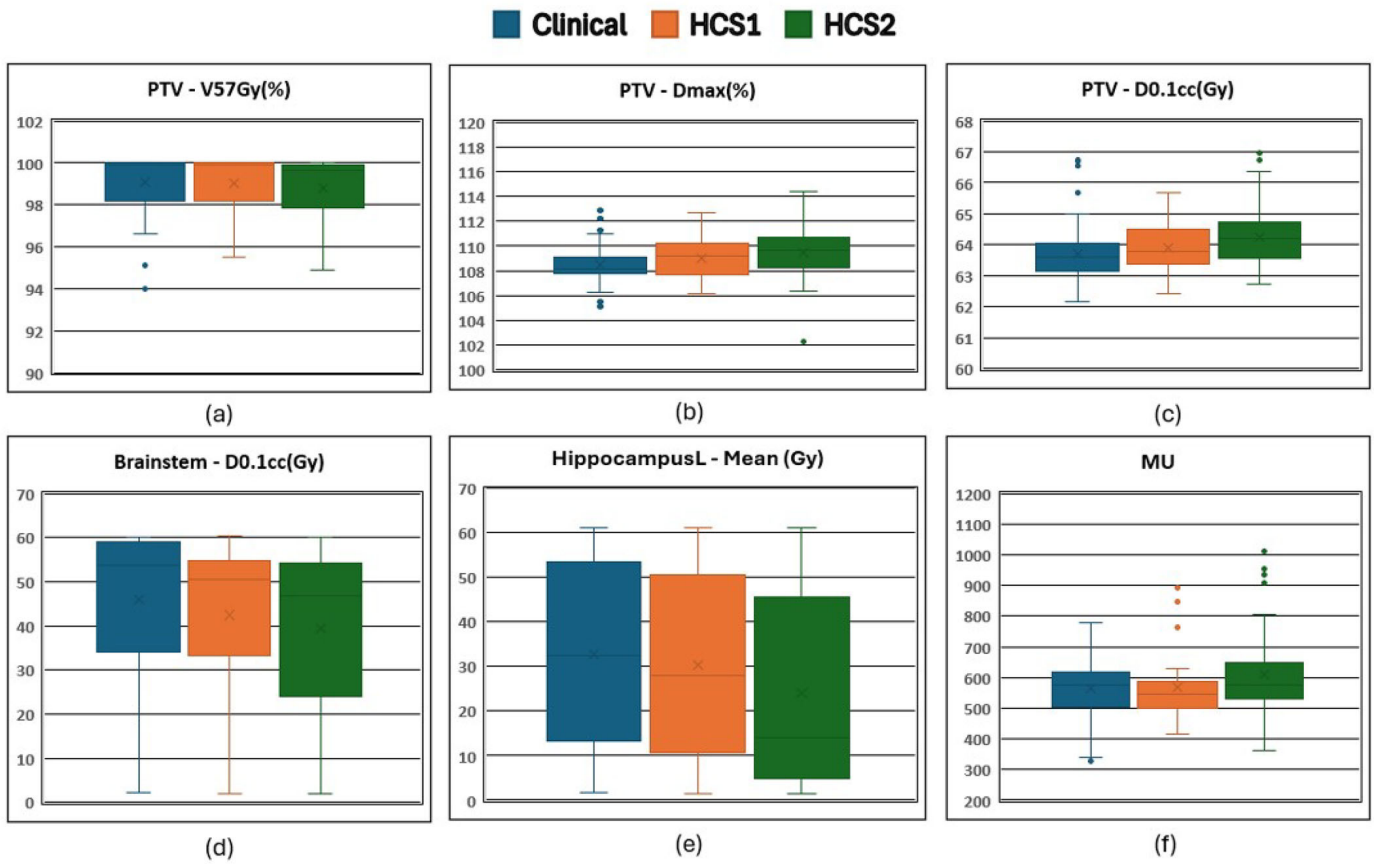
Box and Whisker plots for 50 plans used for validation (a) V57Gy (in %) for PTV, (b) maximum dose of PTV, (c) D0.1cc (in Gy) for PTV, (d) D0.1cc (in Gy) for brainstem, (e) mean dose for left hippocampus, and (f) Total MU delivered for original (blue), HCS1 (orange), and HCS2 (green) plans. MU, monitor unit; PTV, planning target volume.

The summary of the results for the validation dataset (50 patients) is shown as Box and Whisker plots in Figure 3. The left hippocampus mean dose decreased from 32.65 Gy in clinically treated plans to 30.45 Gy in HCS1 plans (mean difference = 2.20 ± 3.07; *p* < 0.001) and 24.04 Gy in HCS2 plans (mean difference = 8.61 ± 6.62; *p* < 0.001). The dose to 0.1 cc of the brainstem, which lies in close proximity to the left hippocampus, decreased from 45.91 Gy in clinically treated plans to 42.44 Gy in HCS1 plans (mean difference = 3.47 ± 5.61; *p* < 0.001) and 39.29 Gy in HCS2 plans (mean difference = 6.62 ± 6.51; *p* < 0.001). The volume of the PTVs in the model was between 95 and 648 cm^3^. The mean V95% of the PTV was 99.08% ± 1.39% in clinically treated plans, 99.03% ± 1.37% in HCS1 plans (mean difference = 0.05 ± 0.58; *p* = 0.415), and 98.80% ± 1.48% in HCS2 plans (mean difference = 0.28 ± 0.58; *p* < 0.001). The maximum dose to the PTV increased from 108.48% ± 1.51% in clinically treated plans to 109.03% ± 1.48% in HCS1 plans (mean difference = −0.55 ± 1.66; *p* = 0.045) and 109.70% ± 1.79% in HCS2 plans (mean difference = −1.02 ± 2.36; *p* = 0.001). Although we found statistically significant differences in the mean V95% of the PTV between plans, these differences were not considered clinically meaningful by the radiation oncologist who assessed the plans. Based on the radiation oncologist review of the final HCS2 plans, 37 of 50 (74%) plans were clinically acceptable for treatment without manual refinement.

**FIGURE 4:**
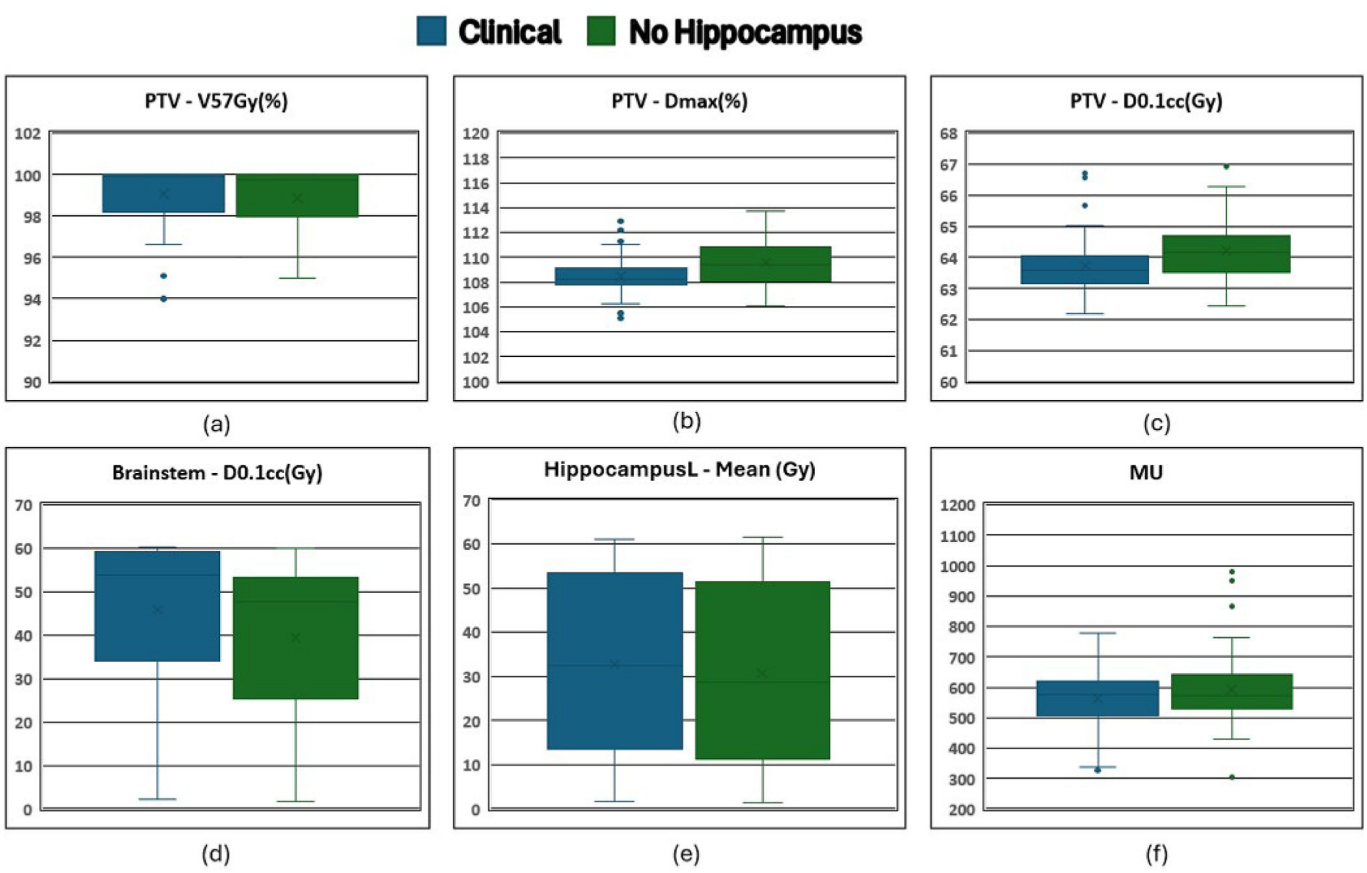
Box and Whisker plots for 50 plans without using the hippocampus as an optimization structure (a) V57Gy (in %) for PTV, (b) maximum dose of PTV, (c) D0.1cc (in Gy) for PTV, (d) D0.1cc (in Gy) for brainstem, (e) mean dose for left hippocampus, and (f) Total MU delivered for original plans (blue) and the plans without the hippocampus contoured (green). MU, monitor unit; PTV, planning target volume.

For the 50 plans not using the hippocampus as an optimization objective, the left hippocampus mean dose still decreased from 32.65 Gy in clinically treated plans to 30.64 Gy in plans without a hippocampus optimization objective (mean difference = 2.01 ± 2.96; *p* < 0.001). The dose to 0.1 cc of the brainstem also decreased from 45.91 Gy in clinically treated plans to 39.41 Gy in plans without hippocampus optimization objective (mean difference = 6.50 ± 6.48; *p* < 0.001). The summary of results for brainstem, left hippocampus, PTV and number of MUs is shown as Box and Whisker plots in Figure 4.

Modulation factors, defined as the total number of MUs per dose per fraction, were also assessed. The modulation factor increased from 2.65 ± 0.46 in clinically treated plans to 2.65 ± 0.28 (mean difference = −0.00 ± 0.44; *p* = 0.811) in HCS1 plans and 2.85 ± 0.34 in HCS2 plans (mean difference = −0.2 ± 0.52; *p* = 0.011). A nominal criterion for acceptable modulation is a factor of less than 4, which was met for all clinically treated, HCS1 and HCS2 plans.

**FIGURE 5:**
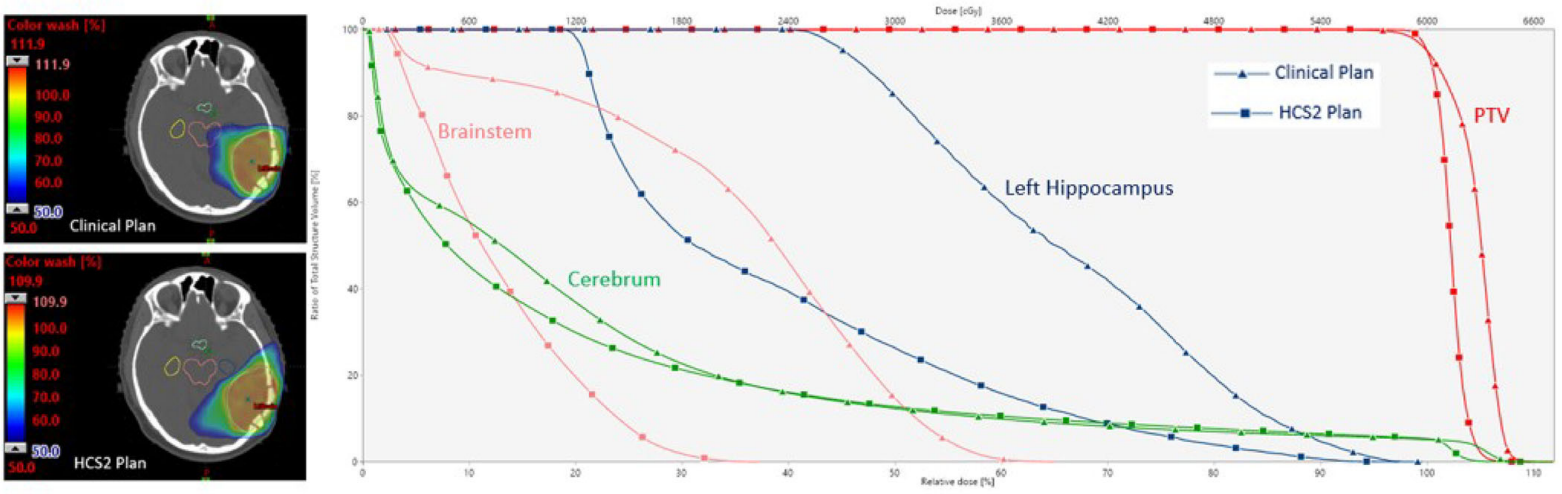
Example of the dose distribution in the clinical plan (top left) and HCS2 plan (bottom left). The brainstem and left hippocampus are shown in these cross sections. The DVH for the PTV (right) including the hippocampus, brainstem, and cerebrum in the clinical plan (triangles) and HCS2 plan (squares). DVH, dose volume histogram; PTV, planning target volume.

An example of the dose distribution at the hippocampus level for a glioblastoma plan is shown in Figure 5. A DVH comparison between the clinically treated and the HCS2 plans for the PTV, left hippocampus, brainstem, and cerebrum structures is shown in Figure 5 for the same patient.

## 4 DISCUSSION

In this planning study using RP with MCO for patients with glioblastoma, mean left hippocampal dose was reduced by 26% compared to clinically treated VMAT plans that did not prioritize hippocampal sparing, without compromise of PTV coverage. The use of RP combined with MCO enabled us to create plans with left hippocampus sparing. The clinical plans that we used to create our initial RP model (HCS1) did not have the left hippocampus as an optimization objective. Applying MCO on HCS1‐generated plans allowed us to investigate the limit on how much we can lower the dose to the left hippocampus while maintaining clinically acceptable target coverage. The final RP model can generate clinically acceptable plans in a single optimization with reduced mean dose to the left hippocampus when compared to the original plan.

The maximum dose to the PTV was typically higher in plans generated using MCO (∼2%) and RP (∼1%). This is not surprising: when exploring the MCO trade‐offs and prioritizing reduction of the mean dose to the left hippocampus, allowing small increases to the PTV maximum dose were required to ensure that the PTV constraints were not otherwise compromised. Nevertheless, the mean maximum dose to the PTV remained below the acceptable protocol limit of 110% of the prescription dose. Although the mean number of MUs was higher in final plans, reflecting an increase of the plan complexity, the modulation factors remained within the range of acceptable values for clinical plans.

The final model also has some benefit even without hippocampus delineation at the time of plan optimization, which can be a useful time saving measure for clinicians. Based on the 50 plans, the mean dose to the left hippocampus was still reduced by 6% compared to the original clinically treated plans. This is expected as the MCO‐generated plans affected the DVHs for the other OARs in a way that spared the left hippocampus, and those DVHs were used to create our final HCS2 model. When the final model is applied to the new data, the DVHs within the model provided left hippocampus sparing even without explicitly specifying optimization objectives for the left hippocampus. In our model, the proximity of the brainstem to the hippocampus is the major influence. As shown in Figure 5, the dose to the brainstem is drastically lower in the RP plan compared to the clinical plan, which also reduces the dose to the hippocampus.

Our final model can be implemented within our clinical workflow for patients diagnosed with glioblastoma whether or not the hippocampus is delineated. Our study does, however, have some limitations. We used brainstem, left hippocampus, and PTV structures in our exploration of the trade‐off when applying MCO. Additional OARs can also be added to the exploration space. This, however, would increase planning time as more plans are created to explore all possible trade‐offs. For our initial goal, our approach was sufficient to improve the quality of the plan to create an RP model that effectively reduced the mean dose to the left hippocampus. The data to create an RP model can be improved in an iterative process, by further refining the quality of data used to train the model. Our planning study used the same hippocampal dose constraint for conventionally fractionated and hypofractionated dose radiotherapy regimens. Biological injury to the hippocampus would be higher at a hippocampal dose of 30 Gy within a hypofractionated regimen, however, compared to a conventionally fractionated regimen. A linear‐quadratic model assuming an α/β ratio of 3 for normal brain consistent with QUANTEC models,^21^ would yield an equivalent dose constraint for the hippocampus of 27 Gy in 20 fractions to 30 Gy in 30 fractions. Neurocognitive outcomes after hippocampal irradiation above and below this hippocampal dose constraint for hypofractionated radiotherapy are not available in the literature, however, and further study is needed. Further study is also needed to investigate the association of the proximity of the PTV to the left hippocampus, which we did not address in the present study.

## 5 CONCLUSIONS

We demonstrated that an improved RP model can be created in a two‐step process using MCO to provide left hippocampus sparing as much as is reasonably achievable while maintaining clinically acceptable PTV and other OAR metrics. Our study demonstrates that the final model can be used to reduce hippocampal dose even if the hippocampus is not contoured and thus not available as an optimization objective. Our two‐step RP model creation strategy provides an efficient and efficacious process to reduce the left hippocampal dose while still meeting other planning objectives, without a requirement for additional planning time spent by radiation oncologists and dosimetrists.

## AUTHOR CONTRIBUTIONS

Shima Y. Tari led conception and design of the project, data analysis and interpretation, RP model creation and validation, and drafted the initial version of the manuscript. Amr Heikel contributed to data acquisition, analysis, and interpretation. Connie Le contributed to data acquisition and reviewed patient's plans. Fan Yang and Deepak Dinakaran contributed to data acquisition and analysis, and reviewed patient's plans. John Amanie, Albert Murtha, Lindsay S. Rowe, and Wilson H. Roa contributed to data acquisition. Samir Patel contributed to design of the project, led data acquisition and interpretation. All authors critically revised the manuscript for important intellectual content, provided final approval of the version to be published, and agree to be accountable for all aspects of the work.

## CONFLICT OF INTEREST STATEMENT

The authors declare no conflicts of interest.

## Data Availability

The data that support the findings of study including RP models are available from the corresponding authors upon reasonable request.
